# Impact of two rounds of azithromycin mass drug administration on active trachoma and ocular *Chlamydia trachomatis* infection in Naoero

**DOI:** 10.1371/journal.pntd.0014679

**Published:** 2026-09-16

**Authors:** Carleigh S. Cowling, Sue-Chen Apadinuwe, Anasaini Cama, Mitchell Starr, Emma M. Harding-Esch, Cristina Jimenez, Ana Bakhtiari, Sarah Boyd, Sara Webster, Anthony W. Solomon, Simon Reid, John M. Kaldor, Susana Vaz Nery

**Affiliations:** 1 The Kirby Institute, UNSW, Sydney, Australia; 2 Ministry of Health and Medical Services, Denig, Naoero; 3 Pacific Eye Care Society, Suva, Fiji; 4 NSW State Reference Laboratory for HIV, St Vincent’s Centre for Applied Medical Research, St Vincent’s Hospital, Sydney, Australia; 5 London School of Hygiene & Tropical Medicine, London, United Kingdom; 6 Sightsavers, Haywards Heath, United Kingdom; 7 International Trachoma Initiative, The Task Force for Global Health, Decatur, Georgia, United States of America; 8 Malaria & Neglected Tropical Diseases Department, World Health Organization, Geneva, Switzerland; 9 School of Public Health, University of Queensland, Herston, Australia; Medical University of Vienna, AUSTRIA

## Abstract

**Background:**

Trachoma, caused by ocular infection with the bacterium *Chlamydia trachomatis*, is the primary infectious cause of blindness globally. To contribute to the elimination of trachoma as a public health problem, Naoero undertook two rounds of antibiotic mass drug administration (MDA) using azithromycin, with population-level coverage estimated at 87% and 93%. This study aimed to estimate the post-MDA prevalence of trachoma, and infection with *C. trachomatis*, in children aged 1–9 years.

**Methods:**

A two-stage cluster sampled survey was conducted 12 months after the second MDA round. All residents aged ≥1 year were examined for signs of trachoma, and conjunctival swabs were collected from children aged 1–9 years for PCR testing. Data on domestic access to water, sanitation, and hygiene services were also collected.

**Results:**

Overall, 2,150 residents were examined, with 724 aged 1–9 years. The age-adjusted prevalence of trachomatous inflammation—follicular (TF) in children aged 1–9 years was 6.0% (95%CI 3.9–7.7). The age-adjusted prevalence of *C. trachomatis* in the same age group was 2.7% (95%CI 0.3–5.1). There was evidence of trachomatous trichiasis (TT) previously unknown to the health system in one resident aged ≥15 years out of 1114 examined (age- and gender-adjusted prevalence 0.04%, 95%CI 0–0.12). Access to improved water sources for hygiene purposes was reported in 99.8% of households surveyed, and 99% of households reported access to an improved latrine.

**Conclusions:**

In Naoero, the impact survey prevalence of TF was above, and of TT was below, the thresholds defined by WHO as indicating “elimination as a public health problem”. The *C. trachomatis* prevalence estimate was lower than it was pre-MDA (39.4%), demonstrating the impact of antibiotic MDA. Despite the potential value of PCR data in addition to clinical indicators to help inform the need for trachoma elimination interventions, particularly in post-MDA or low TF prevalence areas, standardised thresholds for interpreting *C. trachomatis* prevalence have yet to be established. They are urgently needed so that they can be formally used to guide future interventions.

## Author summary

This study looked at the clinical signs of trachoma, the world’s leading infectious cause of blindness, as well as biological evidence of current bacterial *Chlamydia trachomatis* infection, in children in Naoero. The data were collected after two rounds of mass drug administration (MDA) of azithromycin for trachoma. We show that trachoma and *C. trachomatis* prevalence declined post antibiotic treatment, however, trachoma prevalence continues to fulfil the definition of trachoma as a public health problem, suggesting that public health interventions, including MDA with antibiotics and ongoing health promotion and environmental health improvements are required. As global efforts to eliminate trachoma continue, this survey demonstrates that integrating biological analysis into population-based surveys is both feasible and valuable for informing programme decision-making, although internatioally agreed thresholds for interpreting *C. trachomatis* are needed.

## Background

Trachoma, due to ocular infection with the bacterium *Chlamydia trachomatis*, is the primary infectious cause of blindness globally [1,2]. Infection leads to inflammation of the conjunctival tissue of the upper eyelid, manifested as conjunctival follicles and inflammatory thickening, which may be sufficiently florid to merit the diagnosis of the signs “trachomatous inflammation—follicular” (TF) and “trachomatous inflammation—intense” (TI), respectively [3]. With repeated *C. trachomatis* infections, the eyelid can become scarred (“trachomatous scarring”, TS), causing it to contract and distort so that the eyelashes turn inwards and touch the eyeball, a condition known as “trachomatous trichiasis” (TT). TT is associated with abrasion of the cornea, which can lead to vision loss and blindness, generally in adulthood [1,4]. The World Health Organization (WHO) simplified trachoma grading system provides a standardised and user-friendly framework for diagnosing and monitoring the prevalence of trachoma, to enable efficient public health interventions [5,6]. Laboratory detection of *C. trachomatis* infection is undertaken by nucleic acid amplification tests (NAATs), such as polymerase chain reaction (PCR) [4,5]. NAATs, although historically predominantly used in research settings, are now increasingly being incorporated in routine trachoma prevalence surveys to support evidence-based programmatic decision-making [7,8].

The internationally agreed target for trachoma programmes by 2030 is “elimination as a public health problem” [9], defined by a prevalence of TT ‘unknown to the health system’ of less than 0.2% in adults aged 15 years or older; a prevalence of TF of less than 5% in children aged 1–9 years; and evidence that the health system can identify and manage incident cases of TT [10]. WHO, through the WHO Alliance for the Global Elimination of Trachoma, advocates use of the SAFE strategy for trachoma elimination. SAFE has four components, which are Surgery to address TT, Antibiotic mass drug administration (MDA) to clear infection, and the promotion of Facial cleanliness and Environmental improvement to diminish transmission [7,8,11,12]. Decisions regarding initiation and cessation of the A, F and E components of SAFE are guided by the prevalence of TF in children aged 1–9 years. According to current recommendations, MDA is recommended for entire districts where the prevalence of TF in children aged 1–9 years is ≥ 5% [12].

Naoero, previously known as Nauru, is a small Pacific Island country in the Micronesia group, with just over 21km^2^ of land area, home to an estimated population of 11,215 people [13]. In 2019, a baseline prevalence survey found high prevalences of TF (21.8%), *C. trachomatis* infection (34.5%) and anti-*C. trachomatis* antibodies (34.8%) among 1–9-year-olds [14]. After a first round of MDA in June 2020 (87% coverage, based on number treated/estimated population [13]), an impact survey conducted in May 2021 returned a TF prevalence in 1–9-year-olds of 6.2% (S1 Table). Biological samples were not collected for the 2021 survey. A further round of MDA took place in October 2021, with 93% population treatment coverage (estimated as above). Here, we report the results of a second impact survey, undertaken in October to December 2022.

## Methods

### Ethical statement

Ethical approval was obtained by the National Health Research Ethics Committee of Naoero and University of New South Wales (UNSW) Human Research Ethics Committee. Tropical Data support for the surveys was approved by the London School of Hygiene & Tropical Medicine Observational Ethics Committee (16105). Parents/guardians were never asked to leave children alone with study staff, with all staff completing appropriate checks prior to working with children. During the survey, any individual with signs consistent with active trachoma was given tetracycline eye ointment, as per WHO recommendations [12].

We conducted a household prevalence survey using a two-stage cluster sampling design, with the methodology in line with WHO recommendations [15]. All residents of selected households aged 1 year or older were eligible for clinical examination and children aged 1– 9 years were eligible for conjunctival swab collection.

### Sampling approach

The survey was conducted with Tropical Data support [16]. The sample size for the trachoma impact survey was estimated by calculating the single-population-proportion-for-precision formula (n = DEFF x p(1-p)/(2 x d/(1.96 x 2)²)), corrected for a small population, assuming a TF prevalence of 4% ± 2%, and using a standardised design effect (DEFF) of 2.63 [16,17]. Twenty clusters were randomly selected using the 15 administrative districts of Naoero plus an additional 5 subdivisions from the three most heavily populated districts. In each cluster, 25 households were selected using random sampling, using a pre-existing household list held by the Ministry of Health and Medical Services (MOHMS). A household was defined as a group of people living together and/or eating together. Surveys were undertaken by MOHMS personnel after 4 pm on weekdays, when most residents were likely to be at home.

### Training of trachoma impact survey team

Five MOHMS public health workers were trained as graders by a Tropical Data-certified Principal Trachoma Grader Trainer (2019) using the Tropical Data Training System for Trachoma Prevalence Surveys (Version 3 Training Method). Training followed the standard Grader Qualifying Workshop protocol and schedule. All five trainees successfully achieved the required kappa scores in both the Inter-Grader Agreement (IGA) classroom and field assessments.

Recorder training was also conducted in accordance with the Tropical Data Training System for Trachoma Prevalence Surveys (Version 3 Training Method), following the standard Recorder Training Workshop schedule. All five trainees passed the recorder reliability assessment. Training was led by the in-country Tropical Data-certified Recorder Trainer, with support from a Tropical Data-certified Principal Recorder Trainer virtually, due to COVID-19-related travel restrictions.

Field assistants recruited from MOHMS public health services received theoretical and practical, hands-on training in conjunctival swab collection following completion of the grader and recorder workshops, with field teams trained together on standardised procedures. Training focused on maintaining collection techniques to avoid specimen contamination, accurate specimen labelling, and appropriate storage and handling of samples in the field.

### Recruitment and consent of participants

Verbal information about the survey, including its purpose, aims and requirements of participants, was provided to participants and the parents/guardians of those aged <18 years, along with a hard copy participant information statement. A flip chart was developed to assist the consent process. Verbal consent for ocular examination was recorded in the Tropical Data application used for data collection, and written consent from a parent or guardian was required for the collection of a conjunctival swab from each child aged 1–9 years. Involvement in the survey was voluntary, and participants were free to withdraw at any time.

### Water, Sanitation and Hygiene (WASH) data

The head of each household was asked to respond to questions from a standardised questionnaire about access to water and sanitation [18]. Household latrine and handwashing facilities, if present, were inspected to capture details of their availability and type. Water sources were categorised as improved or unimproved as per the WHO/UNICEF Joint Monitoring Programme [19]. A full list of WASH variables can be found in S2 Table.

### Clinical examination

Residents over twelve months of age were examined by trained, Tropical Data-certified graders for TF, TI, and trichiasis (upper and lower eyelid, separately) [16]. For individuals with trichiasis (upper or lower eyelid), the presence or absence of TS was also recorded, and participants were asked if they had ever been offered management for the respective eyelid(s) by a health care worker at a primary, secondary or tertiary health unit. To validate the response to the health management questions, the grader looked for evidence of a surgical scar. Examination followed the standard procedures of the WHO simplified trachoma grading system [3] using binocular magnifying loupes [20], sunlight or a torch for illumination, and follicle size guides to aid the diagnosis of TF [21].

### Conjunctival swab collection and PCR testing

Conjunctival swabs were collected from participants aged 1–9 years whose parents/guardians had provided formal written consent, using the Cobas Media Dual Swab Sample kits (Cobas, Roche Diagnostics, New Jersey, USA). Cobas PCR Media (Cobas, Roche Diagnostics, New Jersey, USA) is designed to preserve and stabilise clinical specimens for up to twelve months when stored at 2–30°C [22]. A polyester-coated cotton swab was passed three times over the left upper tarsal conjunctiva, with a ~ 120° turn between each pass, then placed into a Cobas PCR media tube. The following preventive measures were implemented to minimise the risk of cross-contamination during field specimen collection: ensuring graders cleaned their hands with alcohol hand gel between each participant examined, using swabs that contacted only the conjunctiva, and placing each swab into a tube held by an assistant who sealed it tightly immediately after collection. The assistant was also in charge of the storage and management of the samples.

Swabs were stored in an airconditioned room below 30°C for up to three months in Naoero before being shipped at room temperature to St. Vincent’s Centre for Applied Medical Research in Sydney, then stored at 2–8°C until laboratory testing, in line with Cobas PCR (Cobas, Roche Diagnostics, New Jersey, USA) storage guidelines [22].

*C. trachomatis* DNA extraction from the conjunctival swabs and PCR testing were conducted on the ROCHE Cobas 6800 analyser. The Cobas CT/NG assay simultaneously detects *C. trachomatis* and *Neisseria gonorrhoeae* DNA. As this study focused on trachoma, only the *C. trachomatis* results were included in our analyses. Samples were tested individually, with results categorised as negative or positive according to the manufacturer’s instructions. Laboratory staff were masked to all clinical data, and samples were de-identified.

### Data entry

Demographic, clinical, and WASH data were entered into smartphones in the field, by Tropical Data-trained and certified data recorders, using the Tropical Data app (Version v1.4.7) [16]. Data are owned by the MOHMS.

### Data analysis

Resident, household and laboratory data were linked through unique identifiers. The prevalences of TF and TI among children aged 1–9 years were estimated by calculating age-specific proportions of TF and TI at cluster level and applying weights based on the distribution of each single-year age group in Naoero, as derived from the most recent census data [13]. Likewise, the proportion of individuals aged ≥15 years with TT in each cluster was adjusted by weighting the observed TT prevalence within each gender-specific 5-year age group by the distribution of that age and gender within the local population. The means of the adjusted cluster-level proportions were taken as the prevalence estimates for Naoero. This approach aimed to account for non-random differences in examination availability across age groups and genders, which would otherwise potentially lead to systematic biases in prevalence estimates [17]. Analysis of PCR data was undertaken using the same approach as for TF. Household variables were attributed to each member of the household for association analyses.

Mixed-effects logistic regression was used to test for association between age, gender, and household-level WASH variables and positivity for either TF or *C. trachomatis*, with clusters and households as random effects.

All analyses were carried out using STATA version 17 (StataCorp, College Station, TX, USA).

## Results

**Fig 1:**
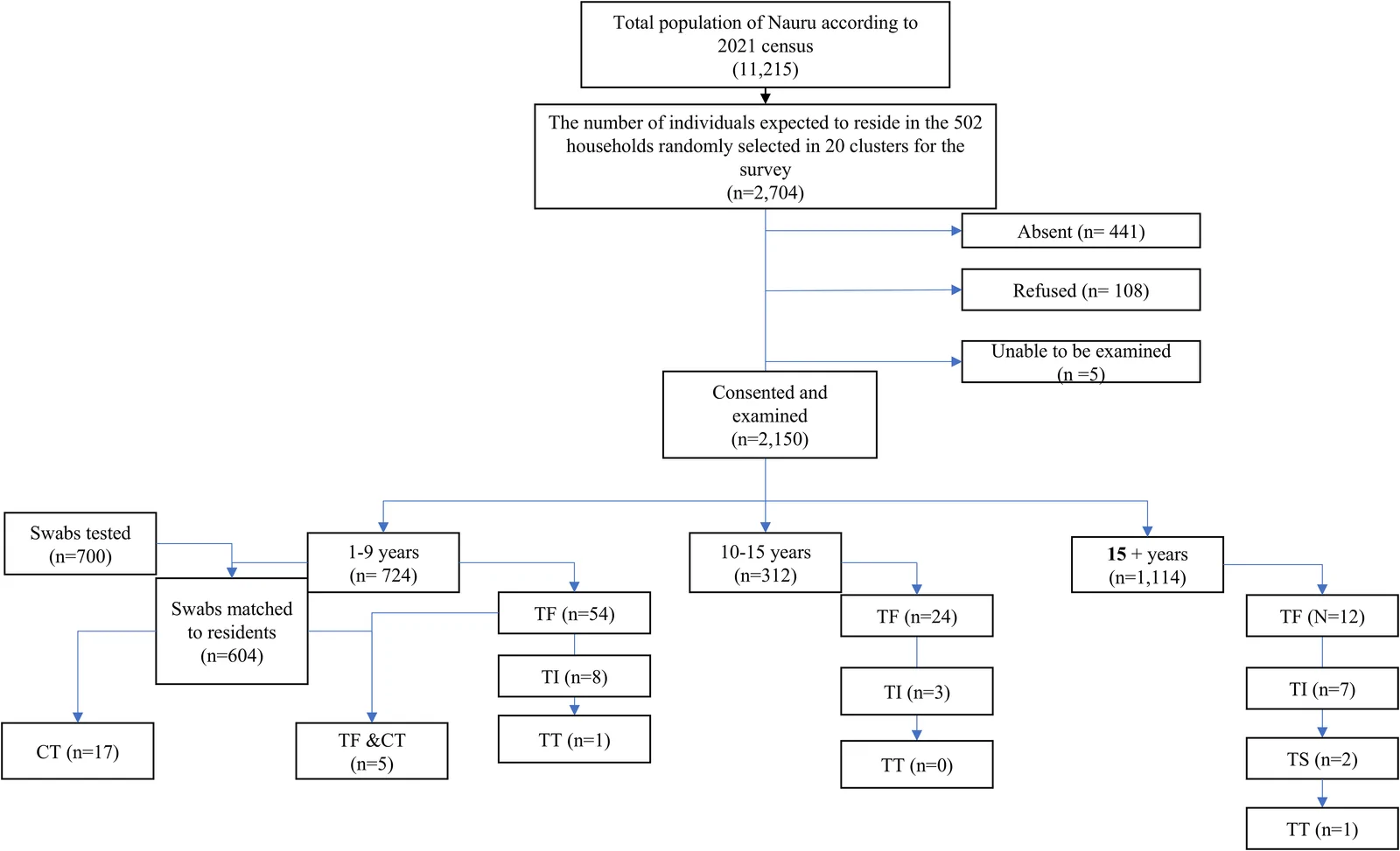
Recruitment of participants and results flow diagram, trachoma impact survey and *Chlamydia trachomatis* infection analysis, Naoero, 2022. TF, trachomatous inflammation—follicular; TI, trachomatous inflammation—intense; TS, trachomatous scarring; Ct, *Chlamydia trachomatis*. * Of 700 samples collected, 96 were unable to be matched due to the unique identifier having not been entered in the Tropical Data app. Amongst these samples, one tested positive.

Overall, 502 households were enrolled across the 20 clusters. Of 2,704 people registered as residents of the enrolled households, 441 (66 aged 1–9 years) were absent at the time the study team visited, 108 (86 aged 1–9 years) refused participation and 5 (3 aged 1–9 years) could not be examined (Fig 1 and Table 1). Of the 2,150 residents examined, 1,200 (56%) were female, 724 (34%) were aged 1–9 years, 312 (15%) aged 10–14 years, and 1,114 (52%) aged ≥15 years, with the oldest resident aged 85 years (Table 1).

**Table 1:** Participation of residents in selected clusters and households surveyed in the trachoma impact survey, Naoero, 2022*.

	1–9 years			10–14 years			15 + years			Overall		
	Male	Female	Total	Male	Female	Total	Male	Female	Total	Male	Female	Total
Residents registered in households and clusters surveyed	442	437	879	184	198	382	629	814	1,443	1,255	1,449	2,704
Examined	363	361	724	141	171	312	446	668	1,114	950	1,200	2,150
	(82.1)	(82.6)	(82.3)	(82.4)	(86.4)	(81.6)	(70.9)	(82.1)	(77.2)	(75.7)	(82.8)	(70.5)
Absent	33	33	66	40	25	65	174	136	310	247	194	441
	(7.4)	(7.5)	(7.5)	(20.4)	(12.6)	(17)	(27.7)	(16.7)	(21.4)	(19.7)	(13.4)	(16.3)
Refused	43	43	86	3	2	5	9	8	17	55	53	108
	(9.7)	(9.8)	(9.8)	(1.6)	(0.1)	(1.3)	(1.4)	(1)	(1.2)	(4.4)	(3.7)	(4)
Other	3		3					2	2	3	2	5
	(0.7)		(0.3)					(0.2)	(0.1)	(0.2)	(0.1)	(0.2)
Tested for *C. trachomatis*	311	293	604		N/A			N/A			N/A	
	(70.3)	(67)	(68.7)									

* The denominator for percentages is residents registered in the households selected for the survey.

### WASH access

All 502 households surveyed provided WASH access data, with 498 households (99%) having access to improved drinking water, and 501 (>99%) having access to improved water for washing (S2 Table). Most households (436 [87%]) had access to a private latrine, while 64 (13%) relied on a shared or community latrine (S2 Table).

### Prevalence of trachoma

Of the 724 children aged 1–9 years examined, 54 (6%, adjusted for age) had TF (95% confidence interval [CI] 3.9–7.7), and 8 (1.0%, adjusted for age) had evidence of TI (95% CI 0.4–1.8) (Table 2).

**Table 2:** Number and age-adjusted prevalence of trachomatous inflammation—follicular (TF), trachomatous inflammation—intense (TI) and *Chlamydia trachomatis* infection (CT) in children aged 1–9 years, Naoero, 2022.

	N	Positive n	Age-adjusted prevalence %	95% CI
**TF**	724	54	6.0	3.9–7.7
**TI**	724	8	1.0	0.4–1.8
**CT**	604	17	2.7	0.3–5.1
CI = Confidence interval.				

**Fig 2:**
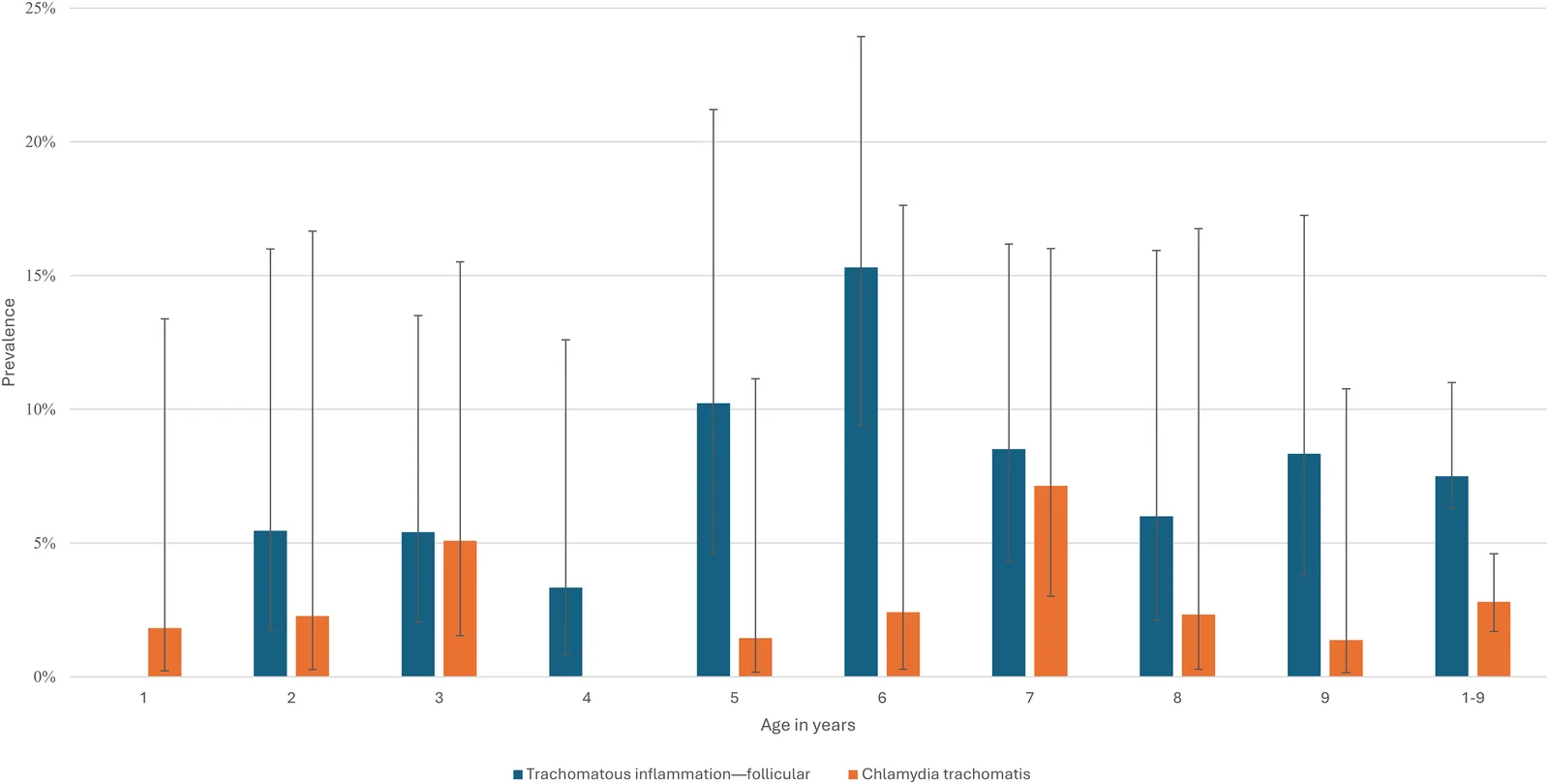
Prevalence of trachomatous inflammation—follicular (TF) and ocular *Chlamydia trachomatis* infection detection by age, in examined children aged 1–9 years, Naoero, 2022.

The proportion of children with TF peaked at age 6 years (Fig 2). For each one-year increase of age, up to the age of 6, there was a 1.17 (95% CI 1.0–1.37, p = 0.048) increase in the odds of having TF (Table 3). There was no evidence of an association between gender and odds of having TF (odds ratio [OR] 0.71, 95% CI 0.33–1.42, p = 0.307) (Table 3).

**Table 3:** Univariable and multivariable model^*^ examining the association between age, gender, source of water for washing, and access to latrines and trachomatous inflammation—follicular (TF), in households with children aged 1–9 years, Naoero, 2022.

Variable	N	TF ^+ ve^ (%)	Univariable model OR (95% CI); p-value	Multivariable model OR (95% CI); p-value
Age, increase per 1 year	724	54 (7.7)	1.17 (1.0–1.37); 0.048	1.17 (0.997–1.36); 0.05
Gender				
Male	363	31 (8.5)	1 (Reference)	
Female	361	23 (6.4)	0.68 (0.33–1.42); 0.307	0.71 (0.34–1.47); 0.356
WASH				
Household water source for washing				
Unimproved	8	2 (25)	1 (Reference)	
Improved	716	52 (7.2)	1.02 (0.004–2.65); 0.170	1.23 (0.05–2.92); 0.680
Access to a latrine				
Private latrine	643	49 (7.6)	1 (Reference)	
Shared/public latrine	79	5 (6.3)	0.86 (0.2–3.73); 0.842	0.89 (0.21–3.73); 0.875
No structure/outside	2	0	N/A	

* Multivariable model includes age, gender, household water source for washing and the place where adults in the household reported defecating, with random intercepts for clusters and households nested within clusters.

CI = Confidence interval.

OR = Odds ratio.

WASH = Water, sanitation and hygiene.

Low TI prevalences were observed in all age groups, with 8 (1.0%; 95% CI 0.4 – 1.8, p = 0.003) children aged 1–9 years, 3 (0.79%; 95% CI 0.3 – 2.4, p = 0.005) individuals aged 10–14 years and 7 (0.49%, 95% CI 0.2 – 1.0, p = 0.002) individuals aged ≥15 years having TI (Tables 2 and S3).

Amongst 1,114 residents aged ≥15 years, 1 (0.04%; 95% CI 0.00 - 0.12, p = 0.002) had TT. This individual was female; her TT was previously unknown to the health system (S3 Table). One child aged six years was also diagnosed with TT unknown to the health system (S3 Table). Neither of the individuals with TT had evidence of TS; although meeting the formal definition of TT [3].

### *Chlamydia trachomatis* infection results

Conjunctival swab PCR results were successfully linked to clinical data for 604 children aged 1–9 years. PCR data were missing for 120 children aged 1–9 years: 24 children refused to be swabbed, and for 96, labelling errors prevented the matching of demographic and PCR data. Of the 604 matched results, 17 were *C. trachomatis* positive, giving an age-adjusted infection prevalence of 2.7% (Table 2). The proportion of children with *C. trachomatis* was highest at age 7 years (Fig 2), but there was no evidence that one-year age increments changed the odds of *C. trachomatis* (OR 0.87, 95% CI 0.56–1.33, p = 0.567)*,* nor was there evidence of gender being associated with having *C. trachomatis* infection (OR 0.54, 95% CI 0.08–3.8, p = 0.538) (Table 4).

**Table 4:** Univariable and multivariable models^*^ examining associations between age, gender, source of water for washing and *Chlamydia trachomatis* infection, in children aged 1–9 years, Naoero, 2022.

Variable	N	*C. trachomatis +*^ve^ (%)	Univariable model OR (95% CI); p-value	Multivariable model OR (95% CI); p-value
Age, increase per 1 year	604	17 (2.8)	0.87 (0.56–1.33); 0.567	0.84 (0.53–1.35); 0.479
Gender				
Male	311	9 (2.7)	1 Reference	
Female	293	8 (2.7)	0.54 (0.08–3.8); 0.538	0.46 (0.52–4.12); 0.491
Household source for washing water				
Unimproved	7	0	N/A	N/A
Improved	597	17 (2.7)		

* Multivariable model includes age and gender, with random intercepts for clusters and household nested within clusters.

CI = Confidence interval.

OR = Odds ratio.

### WASH access

None of the WASH variables had an association with either TF or conjunctival *C. trachomatis* infection in the univariable or multivariable models (Tables 3 and 4).

## Discussion

This survey aimed to inform future public health decisions by estimating the prevalence of both TF and conjunctival *C. trachomatis* infection in children in Naoero after two high coverage rounds of azithromycin MDA. In 2019, the baseline survey found a TF prevalence in 1–9-year-olds of 21.8% [14]. It was anticipated that in Naoero’s relatively small and geographically constrained population with close links to local health workers, a single high-coverage MDA round could lead to interruption of conjunctival *C. trachomatis* transmission, avoiding the need for the recommended three MDA rounds when TF prevalence is ≥ 10% [12]. However, the 2021 impact survey (after that first MDA round) found a TF prevalence in children of 6.2%, still above the 5% threshold. The present (2022) survey estimated the age-adjusted TF prevalence to be 6.0%, indicating elimination as a public health problem has still not been achieved; the population now technically meets the definition for having “persistent TF” [8] (which just requires at least two impact surveys at which the TF prevalence is ≥ 5%, without ever having been <5%). The simultaneously estimated 2022 *C. trachomatis* infection prevalence of 2.7% is, however, considerably lower than that estimated at baseline, when 34.8% of children had *C. trachomatis* [14]. While this finding indicates a substantial impact of MDA on ocular *C. trachomatis* infection in children, the current elimination criteria are based on TF, and there is no formal guidance on the interpretation of infection prevalence for programmatic decision making.

Nevertheless, it is becoming clear that the use of TF alone as in indicator of trachoma as a public health problem in certain settings, such as post-MDA, has major limitations. Several factors contributing to the difficulties in interpretation of TF prevalence, including its persistence after infection has resolved [23–28], its absence in early infection [23,26,27], and the presence of other agents that can produce a TF-like phenotype [1,29–32]. Thus, unnecessary MDA rounds may be distributed, if, as currently, decisions for A, F and E implementation for trachoma elimination are solely based on TF thresholds. Unintended consequences of MDA implementation may include the emergence of antibiotic resistance in non-*C. trachomatis* infections [33,34], as well as the economic cost and burden of undertaking MDA where funds may be better directed to other activities. Given these limitations, there is a critical need to complement clinical assessments with biological markers in trachoma surveys. Serological analysis that detects *C. trachomatis* anti-Pgp3 antibodies is also increasingly recommended for monitoring exposure to infection. It can be particularly valuable in settings where TF appears to be persisting, to determine whether its presence is indeed due to infection with *C. trachomatis.* The use of both serological and infection markers in guiding programmatic decision-making will in the future be facilitated by the adoption of agreed thresholds [35].

Following review of the survey findings, MOHMS elected to implement a third round of azithromycin MDA in Naoero. This decision was informed by the TF prevalence remaining above the WHO elimination threshold following two rounds of MDA, together with evidence of ongoing *C. trachomatis* transmission. While the *C. trachomatis* infection prevalence observed in this study does not necessarily inform programmatic thresholds for intervention, our data contribute to the growing evidence base needed to better understand the relationship between clinical and infection indicators and to inform future refinement of trachoma surveillance and intervention strategies.

It was not surprising that there was no association between either TF or *C. trachomatis* infection and WASH variables. There is a high level of access to improved WASH infrastructure in Naoero an households, with >99% of households surveyed having an improved source of water for washing and 99.7% having direct access to an improved latrine (either private or shared). In such high access settings, it may be more relevant to investigate the association between WASH-related behaviours and trachoma [36–38], an area of research that remains under methodological development [39,40].

The estimated prevalence of TT unknown to the health system in ≥15-year-olds was low, having fallen from 0.33% (95% CI 0.00–0.88%) at baseline to 0.04% (95% CI 0.00–0.12, p = 0.002) in this survey [14]. This reflects a concerted effort to deliver TT surgery to those needing it in Naoero between these surveys. Neither of the individuals with TT had TS; although meeting the formal definition of TT [3], their trichiasis may have had a non-trachomatous cause.

A limitation of our study was the proportion (14%) of samples that could not be matched to participant-level data due to labelling errors. Of the 96 unmatched samples, however, only one (1%) was positive for *C. trachomatis* (S4 Table). Therefore, it is unlikely that the unmatched samples would have considerably altered the overall age-adjusted prevalence. Review of mismatched or missing data indicated that they were distributed across 18 of the 20 surveyed clusters and involved multiple field teams, rather than being concentrated within a particular team or time period. This pattern suggests that the errors were likely the result of sporadic human error occurring during specimen collection and data recording under field conditions. Nevertheless, this experience highlights opportunities to further strengthen field procedures. Although team members received comprehensive role-specific training and practical training together before data collection, allocating additional time for integrated team-based practice may help reinforce individual responsibilities, specimen handling workflows, and verification steps before data collection. A further limitation of this study is that swabs were collected from only the left upper tarsal conjunctiva. Although this standardised approach allows for comparability between surveys, and limits the burden on the participants, unilateral sampling may miss infections present only in the contralateral eye, particularly for those with low organism loads [41]. Therefore, the prevalence of *C. trachomatis* infection reported here may represent a conservative estimate of ocular infection.

In conclusion, while interventions including MDA in Naoero have substantially reduced TF and *C. trachomatis* infection prevalence, TF prevalence remains above the elimination threshold, potentially highlighting the limitation of using TF as a sole indicator of programme success. National trachoma programmes and global partners should consider utilising biological indicators to complement clinical signs for decision-making. However, the absence of defined thresholds for current or previous *C. trachomatis* infection is a gap in current trachoma programme guidance, limiting the integration of biological data into decision-making processes. Correcting this could help to avoid unnecessary antibiotic use and support countries to achieve trachoma elimination sooner. This may be especially pertinent for small island nations like Naoero, where persistent TF, constrained resources, and unique geographic and demographic factors require tailored public health responses.

## Supporting information

S1 Table Number and proportion of children aged 1–9 years with positive results for trachomatous inflammation—follicular (TF) in Naoero, May 2021. (DOCX)

S2 Table Water, sanitation and hygiene data collected in trachoma impact survey in Naoero, 2022. (DOCX)

S3 Table The number and adjusted prevalence of trachomatous inflammation—follicular (TF), trachomatous inflammation—intense (TI) and trachomatous trichiasis (TT) previously unknown to the health system, in residents surveyed in the trachoma impact survey, by age group, in Naoero, 2022. (DOCX)

S4 Table Number and proportion of unmatched PCR samples that tested positive for *C. trachomatis.* (DOCX)
