# Supplementary material for: Impact of two rounds of azithromycin mass drug administration on active trachoma and ocular Chlamydia trachomatis infection in Naoero
Source: PLoS Negl Trop Dis. 2026 Sep 16;20(9):e0014679. doi: 10.1371/journal.pntd.0014679 (PMC13581005; doi:10.1371/journal.pntd.0014679)
Supplement: Supplementary file 1 [file pntd.0014679.s001.docx]

Supplementary Table 1. Number and proportion of children aged 1-9 years with positive results for trachomatous inflammation—follicular (TF) in Nauru, May 2021.

|  | N | Positive n | Positive % | 95% CI (%) |
| --- | --- | --- | --- | --- |
| TF | 689 | 49 | 6.2 | 2.7 -9.5 |
