# Supplementary material for: Impact of two rounds of azithromycin mass drug administration on active trachoma and ocular Chlamydia trachomatis infection in Naoero
Source: PLoS Negl Trop Dis. 2026 Sep 16;20(9):e0014679. doi: 10.1371/journal.pntd.0014679 (PMC13581005; doi:10.1371/journal.pntd.0014679)
Supplement: Supplementary file 2 [file pntd.0014679.s002.docx]

Supplementary Table 2. Water, sanitation and hygiene data collected in trachoma impact survey in Nauru, 2022

| Variable | Number | Proportion |
| --- | --- | --- |
| **Drinking water source** |  |  |
| Piped water into dwelling | 160 | 31.9 |
| Piped water into yard | 96 | 19.1 |
| Protected dug well | 1 | 0.2 |
| Protected spring | 2 | 0.4 |
| Rainwater collection | 135 | 26.9 |
| Water vendor | 78 | 15.5 |
| Bottled water | 26 | 5.2 |
| Other | 4 | 0.8 |
| **Time to get drinking water** |  |  |
| Water source in yard | 400 | 79.7 |
| Less than 30 minutes | 88 | 17.5 |
| 30 minutes to 1 hour | 14 | 2.8 |
| **Washing water source** |  |  |
| Piped water into dwelling | 169 | 33.7 |
| Piped water into yard | 81 | 16.1 |
| Protected dug well | 59 | 11.8 |
| Unprotected dug well | 1 | 0.2 |
| Protected spring | 4 | 0.8 |
| Rainwater collection | 105 | 20.9 |
| Water vendor | 81 | 16.1 |
| Bottled water | 2 | 0.4 |
| **Time to get washing water** |  |  |
| Water source in yard | 423 | 84.3 |
| Less than 30 minutes | 72 | 14.3 |
| 30 minutes to 1 hour | 7 | 1.4 |
| **Where adults defecate** |  |  |
| Private latrine | 436 | 86.9 |
| Shared/public latrine | 64 | 12.8 |
| No structure/outside | 2 | 0.4 |
| **Where faeces of child under 3 years is disposed** |  |  |
| Child uses toilet/latrine | 53 | 10.6 |
| Put into toilet/latrine | 4 | 0.8 |
| Thrown into garbage | 203 | 40.4 |
| Don't know | 1 | 0.2 |
| Other | 2 | 0.4 |
| No child under 3 in household | 239 | 47.6 |
| **Handwashing facility in the yard/plot/premises** |  |  |
| No | 9 | 1.8 |
| Yes | 493 | 98.2 |

* Washing for face, hands, and body hygiene.
