# Supplementary material for: Impact of two rounds of azithromycin mass drug administration on active trachoma and ocular Chlamydia trachomatis infection in Naoero
Source: PLoS Negl Trop Dis. 2026 Sep 16;20(9):e0014679. doi: 10.1371/journal.pntd.0014679 (PMC13581005; doi:10.1371/journal.pntd.0014679)
Supplement: Supplementary file 3 [file pntd.0014679.s003.docx]

Supplementary Table 3. The number and adjusted prevalence of Trachomatous inflammation – follicular (TF), Trachomatous inflammation – intense (TI) and Trachomatous trichiasis (TT) previously unknown to the health system, in residents surveyed in the trachoma impact survey, by age group, in Nauru, 2022

|  | 1 - 9  (n =724) | | | 10 - 14  (n=312) | | 15 +  (n =1,114) | | Total  (n =2,150) | |
| --- | --- | --- | --- | --- | --- | --- | --- | --- | --- |
|  | Number (%) | | SE  (95% CI) | Number (%) | SE  (95% CI) | Number  (%) | SE  (95% CI) | Number  (%) | SE  (95% CI) |
| TF | 54  (6) | 0.009  (4.6–7.2) | | 24  (7.69) | 0.015  (5.2 - 11) | 12  (1.08) | 0.003  (0.6 – 1.9) | 90 (4.19) | 0.004  (3.4 – 5.1) |
| TI | 8  (1.0) | | 0.003  (0.4–1.8) | 3  (0.79) | 0.005  (0.3 – 02.4) | 7  (0.49) | 0.002  (0.2 – 1.0) | 18 (0.67) | 0.002  (0.4 – 1.1) |
| TT | 1  (0.11) | | 0.001  (0.99 - 1) | 0 | N/A | 1  (0.04) | 0.002  (0.00 - 0.12) | 2 (0.09) | 0.0009 (0.7 -0.5) |
