# Supplementary material for: Impact of two rounds of azithromycin mass drug administration on active trachoma and ocular Chlamydia trachomatis infection in Naoero
Source: PLoS Negl Trop Dis. 2026 Sep 16;20(9):e0014679. doi: 10.1371/journal.pntd.0014679 (PMC13581005; doi:10.1371/journal.pntd.0014679)
Supplement: Supplementary file 4 [file pntd.0014679.s004.docx]

Supplementary Table 4. Number and proportion of unmatched PCR samples that tested positive for *C. trachomatis*

|  | N | Positive n | Positive % | 95% CI |
| --- | --- | --- | --- | --- |
| CT | 96 | 1 | 1 | 0.14 – 7.2 |
